# Epigenetic regulation of embryonic ectoderm development in stem cell differentiation and transformation during ontogenesis

**DOI:** 10.1111/cpr.13413

**Published:** 2023-02-01

**Authors:** Liuyan Huang, Feifei Li, Ling Ye, Fanyuan Yu, Chenglin Wang

**Affiliations:** ^1^ State Key Laboratory of Oral Diseases & National Clinical Research Center for Oral Diseases West China Hospital of Stomatology, Sichuan University Chengdu China; ^2^ Department of Endodontics West China Hospital of Stomatology, Sichuan University Chengdu China

## Abstract

Dynamic chromatin accessibility regulates stem cell fate determination and tissue homeostasis via controlling gene expression. As a histone‐modifying enzyme that predominantly mediates methylation of lysine 27 in histone H3 (H3K27me1/2/3), Polycomb repressive complex 2 (PRC2) plays the canonical role in targeting developmental regulators during stem cell differentiation and transformation. Embryonic ectoderm development (EED), the core scaffold subunit of PRC2 and as an H3K27me3‐recognizing protein, has been broadly implicated with PRC2 stabilization and allosterically stimulated PRC2. Accumulating evidences from experimental data indicate that EED‐associating epigenetic modifications are indispensable for stem cell maintenance and differentiation into specific cell lineages. In this review, we discuss the most updated advances to summarize the structural architecture of EED and its contributions and underlying mechanisms to mediating lineage differentiation of different stem cells during epigenetic modification to expand our understanding of PRC2.

## Graphical abstract

Polycomb repressive complex 2 (PRC2) is composed of four core subunits – embryonic ectoderm development (EED), enhancer of zeste homologue 2, suppressor of Zeste 12, and RB‐binding protein 4/7. PRC2 catalyses methylation of H3K27, an enzymatic activity necessary for PRC2‐mediated epigenetic gene silencing during stem cell differentiation and transformation. PRC2 absent EED is incapable of recognizing binding sites to promote deposition of H3K27me3, results in a significant upregulation of genes that are normally associated with H3K27me marks.

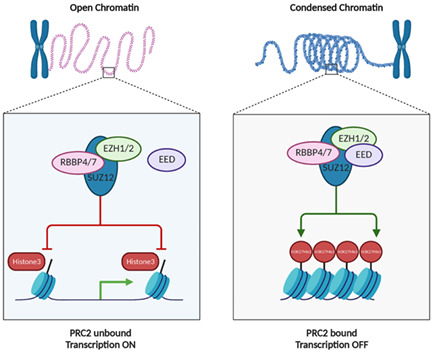

## 1 INTRODUCTION

Organ morphogenesis and functional maturation involve a series of lineage choice and cell‐fate decisions, which require tight spatial and temporal regulation of gene expression, activating the transcription of lineage‐specific transcriptional programmes and simultaneously repressing lineage‐inappropriate gene expression. The nucleosome represents the smallest repeated structural unit of eukaryotic chromatin, consisting of DNA wrapping around histones,^1^, ^2^ while transcription factor (TF) specificity and chromatin accessibility are affected by epigenetic mechanisms.^3^

**FIGURE 1:**
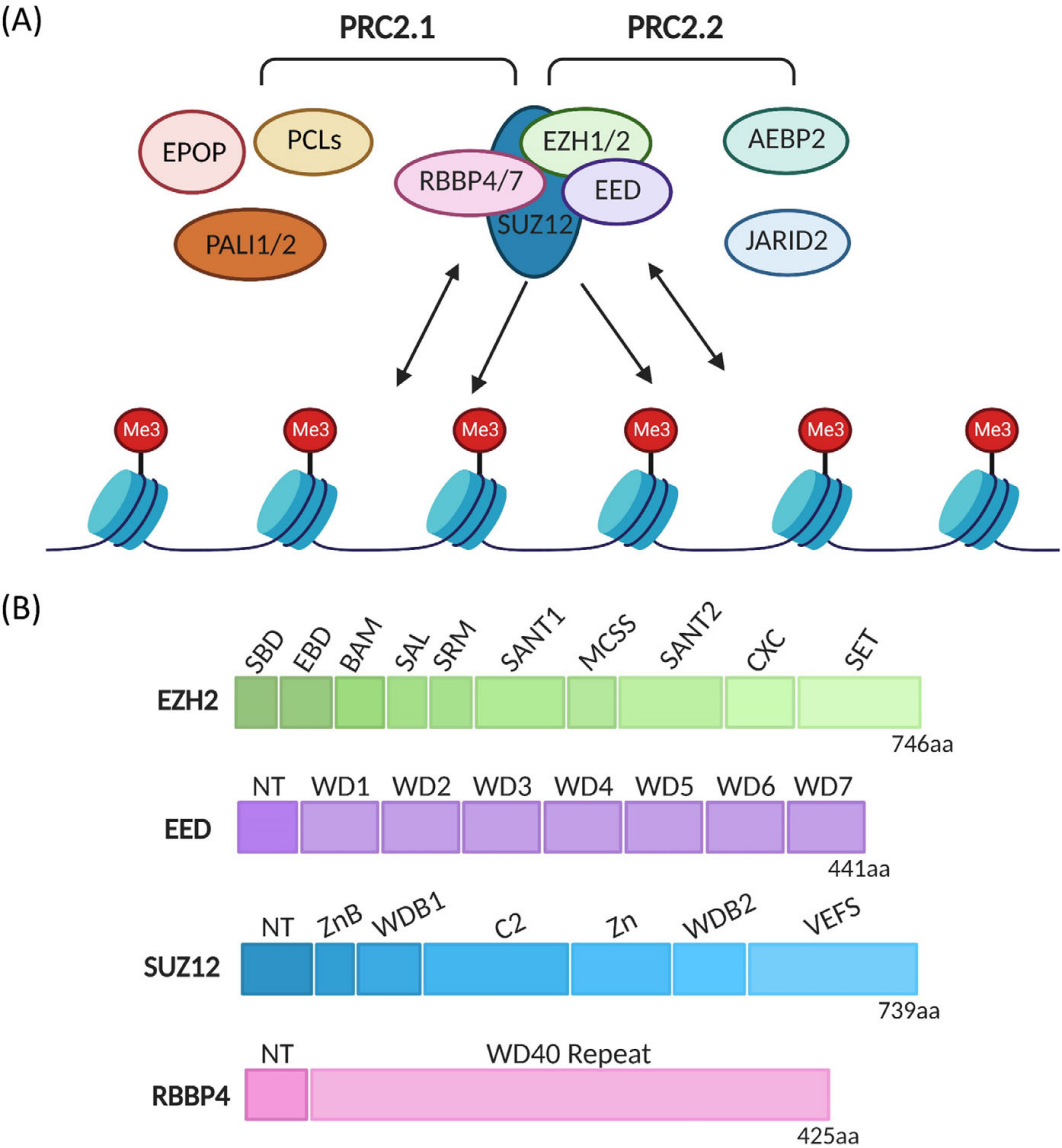
Subunits composition, chromatin association and protein domain structure of Polycomb repressive complex 2 (PRC2). (A) Schematic drawing of PRC2.1 and PRC2.2 core subunits and accessory subunits. Single‐headed arrows represent the deposition of histone marks, and double‐headed arrows depict chromatin binding. (B) Domain structure of PRC2 core subunits. Linker regions are omitted.

**FIGURE 2:**
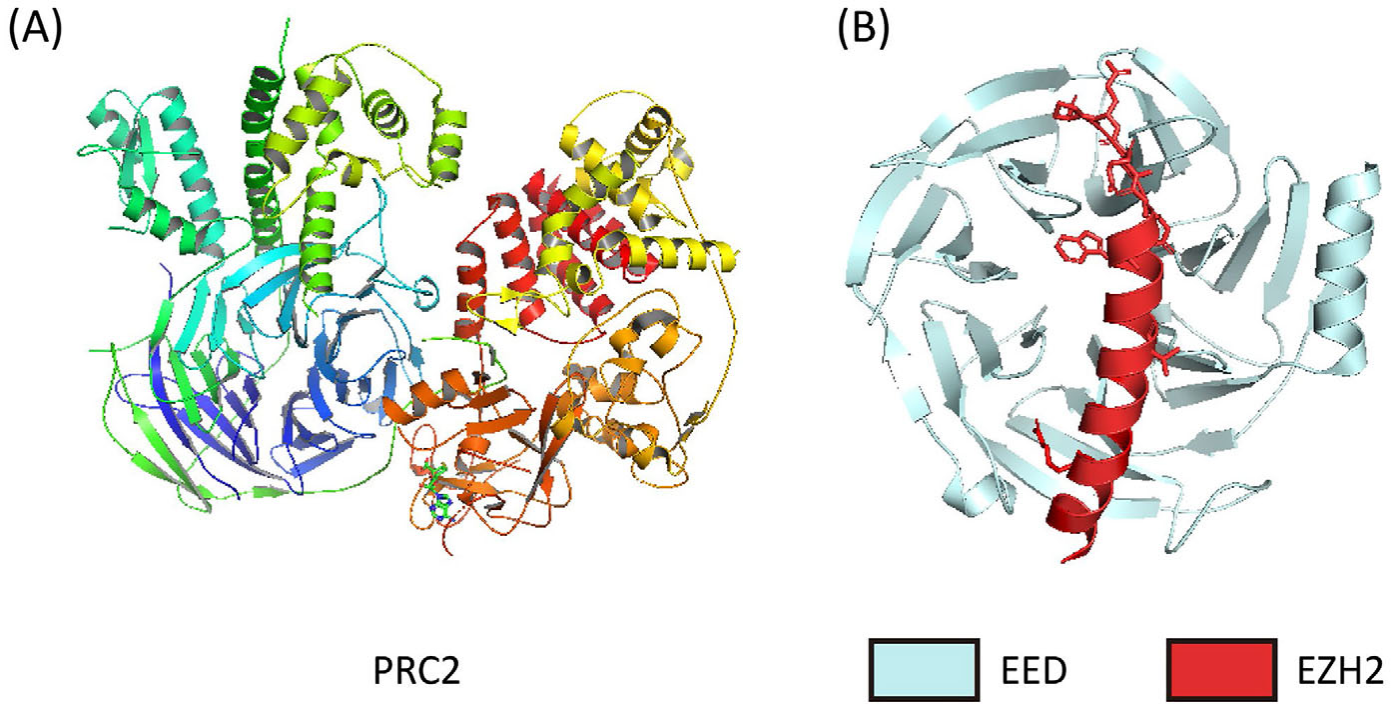
(A) Crystal structure of the Polycomb repressive complex 2 (PRC2) in the basal state. Components of structures are colour‐coded. Structure figure is rendered in PyMOL. PDB ID:5KJI.155. (B) Structure showing embryonic ectoderm development (EED) in complex with the N terminus of EZH2. Structure figure is rendered in PyMOL. PDB ID:2QXV.44.

Polycomb group (PcG) proteins are key epigenetic regulators that mediate heritable transcriptional silencing by modifying chromatin states and participating in the establishment and maintenance of cell fates during multicellular development.^4^, ^5^, ^6^, ^7^, ^8^ They are identified originally in *Drosophila* as repressors of Hox genes and are gradually found in other species, such as human, mouse, *Xenopus*, and so on.^9^, ^10^ PcG proteins have garnered attention for their ability to modulate mammal stem cell differentiation. Generally, there are two types of polycomb proteins in mammals, polycomb repressive complexes 1 and 2 (PRC1 and PRC2), catalysing the ubiquitylation of histone 2A at lysine‐119 (H2AK119ub) and the trimethylation of histone 3 at lysine‐27 (H3K27me3), respectively.^11^ PRC2 is composed of four core subunits, enhancer of zeste homologue 1/2 (EZH1/2), embryonic ectoderm development (EED), suppressor of Zeste 12 (SUZ12) and RB‐binding protein 4 or 7 (RBBP4/RBAP48 or RBBP7/RBAP46).^7^ EZH1/2 are homologous analogs, and EZH1 can partially compensate for EZH2 function in some developmental cells.^12^, ^13^, ^14^, ^15^ The SET‐domain containing protein EZH2 endows the Polycomb PRC2 complex with histone lysine methyltransferase activity, but its activity requires the participation of the other three subunits of the core complex.^16^, ^17^ EZH2 displays an autoinhibited state and exhibits little histone methyltransferase activity when on its own.^18^ In mammals, The WD‐repeat‐containing protein EED is present as four distinct isoforms, which are believed to be mediated by utilizing four in‐frame start codons of a single EED mRNA transcript.^19^ One common critical function of the EED isoforms is stabilizing EZH2 in the PRC2 complex. In addition, the PRC2 complex binds to H3K27me3 through the aromatic cage of EED, which has specific recognition of defined (repressive) trimethylated‐lysine residues.^20^, ^21^ SUZ12 stabilizes PRC2 by interacting with EZH2 via its VRN2‐EMF2‐FIS2‐Suz12 box (VEFS) domain.^22^, ^23^ Furthermore, SUZ12 is responsible for the methylation of lysine 9 of histone 3.^24^ As the fourth core subunit of PRC2, the histone‐binding protein RbAp46/48 is essential for PRC2 binding with histone tails.^25^ Recruitment of the PRC2 complex to chromatin is mediated by interaction with non‐core subunits such as Zinc finger protein AE binding protein 2 (AEBP2), Polycomb‐like homologues (PCLs), PRC2‐associated LCOR isoform 1 or 2 (PALI1/2), Polycomb repressive complex 2‐associated protein (EPOP), and Jumonji and AT‐rich interaction domain containing 2 (JARID2).^7^, ^26^, ^27^, ^28^, ^29^ Non‐core subunits compete to bind to the N‐terminal region of SUZ12, which defines the PRC2.1 and PRC2.2 subcomplexes. PRC2.1 contains one of the three PCLs with either EPOP or PALI1/2, while PRC2.2 contains AEBP2 and JARID2 (Figures 1 and 2A).^28^, ^29^, ^30^, ^31^

A vast number of studies report that the core subunits of the PRC2 complex show spatiotemporal specific expression patterns, indicating that they may have distinct functions.^32^, ^33^, ^34^, ^35^ EZH2 is highly expressed in proliferating cells, while EZH1 is at high levels in the differentiated tissues.^15^, ^36^ EED is generally regarded as a gene silencing regulator that maintains pluripotency of embryonic stem cells (ESCs) and cell proliferation. Furthermore, increasing evidence suggests that EED contributes to stem cell maintenance and lineage specification in ontogenesis.^34^, ^37^, ^38^, ^39^, ^40^, ^41^

This review provides a comprehensive overview of EED in regulating stem cell fate and the underlying regulatory mechanisms during various organ morphogenesis, refining the framework for understanding the functions and mechanisms of PRC2‐mediated regulation of gene expression.

## 2 THE STRUCTURAL BASIS OF EED

As a WD‐40 repeat family protein, EED contains seven WD40‐repeat motifs at its C terminus preceded by an extended N‐terminal segment.^20^ A WD40 repeat also called a WD or β‐transducin repeat is a short, ∼40‐residue motif.^42^, ^43^ The WD40 repeats of EED were multimerized to fold into a canonical seven‐blade β‐propeller structure. The seven blades are radially arranged around a central axis to form a peptide‐binding pocket in the centre of the β‐propeller structure.^20^ EED interacts with the N‐terminal domain of EZH2 through its larger bottom surface of WD40 repeat motifs, which in turn modulates EZH2's histone methyltransferase activity.^44^ An aromatic cage is a common trait for most methyllysine‐binding motifs,^45^ and EED recognizes H3K27me3 through the aromatic cage located on the top surface of its WD40 repeat domain.^46^, ^47^ Through the trimethyllysine, the histone peptide binds to EED and is recognized by the aromatic cage to form EED–H3K27me3 peptide complex structure.^20^ Thus, EED acts not only as a critical molecule to compose the PRC2 complex but also as an ‘epigenetic exchange factor’ to modulate methylation on histones.

## 3 EED MODULATES ALLOSTERIC ACTIVATION AND RECRUITMENT OF PRC2

**FIGURE 3:**
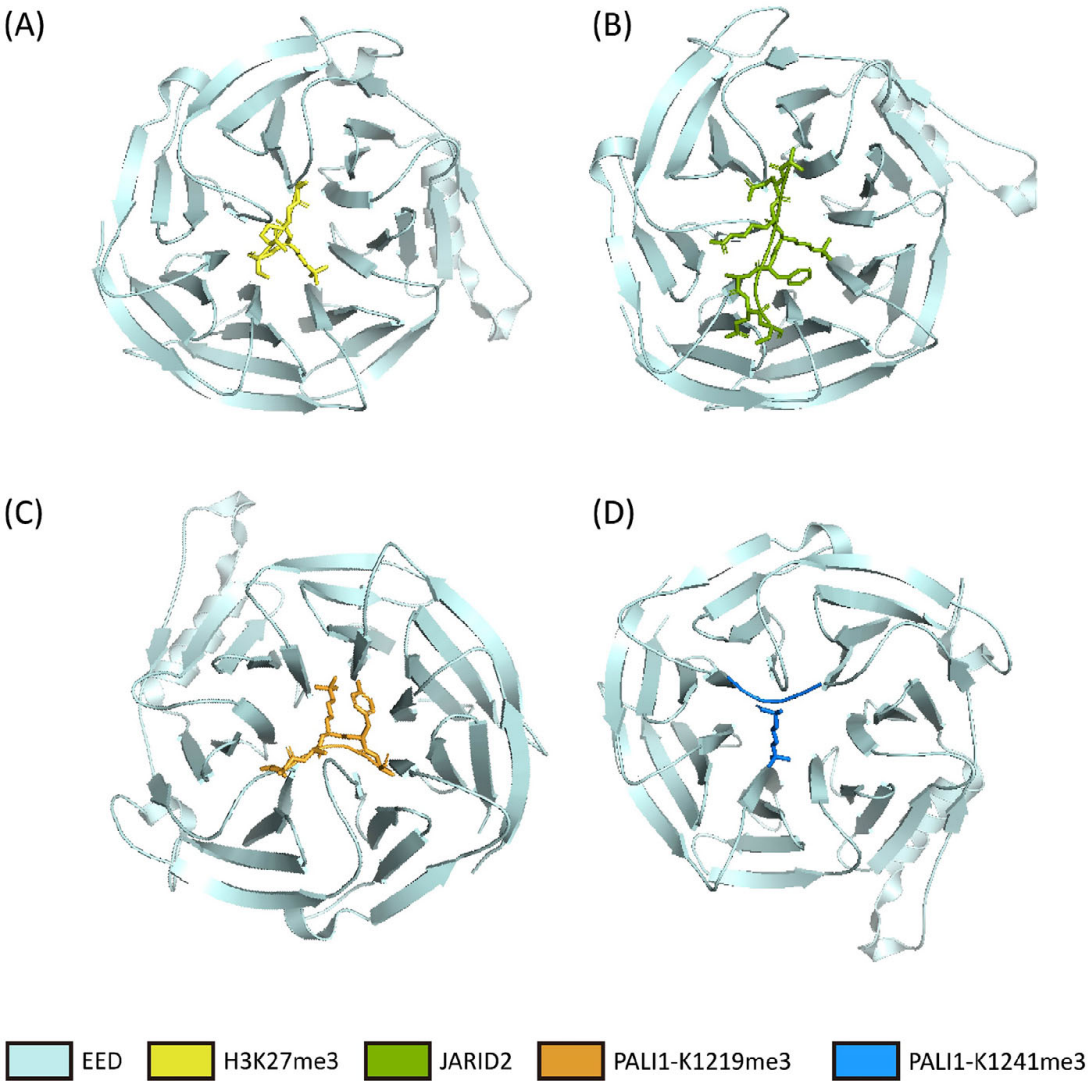
(A) Structure showing embryonic ectoderm development (EED) in complex with H3K27me3. Structure figure is rendered in PyMOL. PDB ID:3JZG.48. (B) Structure showing EED in complex with JARID2. Structure figure is rendered in PyMOL. PDB ID:4X3E.49. (C) Crystal structure of EED in complex with PALI1‐K1219me3 peptide. Structure figure is rendered in PyMOL. PDB ID:6V3Y65. (D) Crystal structure of EED in complex with PALI1‐K1241me3 peptide. Structure figure is rendered in PyMOL. PDB ID:6V3X.65.

EZH2 adopts an autoinhibitory conformation through crystal structures of the inactive isolated catalytic domain, suggesting that structural rearrangement of EZH2 is likely required for PRC2 activation.^18^, ^47^ Moreover, a range of crystallographic structures of EED in complex with EZH2, trimethylated histone and nonhistone peptides highlight the pivotal role of EED in mediating EZH2 binding and triggering PRC2 an allosteric activation of catalysis.^20^, ^44^, ^48^, ^49^ After PRC2 complex assembly, the activation loop from the N‐terminal portion of EZH2 is moved by EED to the neighbouring catalytic SET domain (Figure 2B).^45^ The EED‐binding domain (EBD) of EZH2 occupies the bottom face of the EED WD40 domain, and then three β strands are added to EED to form the β‐addition motif (BAM) and maintain EED in a stable position to allow H3K27me3 binding to the top WD40‐repeat domain of EED (Figure 3A).^47^, ^50^ The SET activation loop (SAL) is formed after the BAM migrates away from the EED surface to the back of the SET domain of the catalytic moiety. Then the H3K27me3 peptide is sandwiched between EED and an exposed EZH2 motif, referred to as the stimulation‐responsive motif (SRM), and immediately follows the SAL of EZH2. SRM of the sandwich‐like assembly interacts extensively with two others and transforms itself into a fully ordered α‐helix‐loop structure.^47^ Finally, the interaction between SAL and the newly formed SRM helix of EZH2 stabilizes the active conformation of the EZH2 SET, resulting in enhanced histone methyltransferase activity of PRC2. In addition, EED along with the SAL and SET regions of EZH2 get glued together by the N‐terminal loop region of SUZ12 (VEFS). Additionally, the SANT1‐binding domain (SBD) of EZH2 contacts with the DNA in the H3K27me3‐marked nucleosome after binding by EED.^47^, ^51^ Through a highly complex series of EED, EZH2, and H3K27me3 interactions, EED allosterically regulates PRC2 histone methyltransferase activity and PRC2 on‐chromatin spreading.^20^, ^52^, ^53^

The PRC2 crystal structures provide fundamental insights into the interaction with each subunit, substrate recognition, and allosteric activation of its enzymatic activity. Moreover, PRC2 is more active on di‐nucleosomes and higher‐order chromatin structures than on mononucleosomes or histone tails,^50^, ^54^, ^55^ indicating the crucial role of interaction with the chromatin in PRC2 activation. A response to allosteric stimulation of PRC2 results in it catalysing H3K27me3 on neighbouring nucleosomes, causing the formation of broad H3K27me3 domains.^56^ Recently, a cryo‐EM structure of PRC2 in di‐nucleosomes revealed that EED engages with one H3K27me3‐modified nucleosome to allow the H3K27me3 thread into the aromatic cage of EED and locates the SET domain present in EZH2 to methylate an unmodified H3 tail on the other adjacent nucleosome.^50^ EED and EZH2 consist of the dominant interface to interaction with di‐nucleosome, while SUZ12, RbAp46/48, AEBP2 and JARID2 could also potentially assist nucleosome interaction.^57^ These structures provide additional mechanistic explanations for the PRC2 complex interacting predominantly with the nucleosome, which is quite different from most other chromatin modifiers interacting with DNA and the conserved histone acidic patch surface.

PRC2 recruitment is modulated by many different factors, including the interaction of PRC2 subunits with DNA and histones, PRC1‐mediated H2AK119ub1, RNA and other histone modifications.^58^, ^59^ PRC2 recruitment involves two main functional axes, one is MTF2‐PRC2.1 binding to DNA, and the other is JARID2 binding to the marker H2AK119ub1, from PRC1.^60^ They are both reinforced by H3K27me3‐EED‐positive feedback. JARID2 incorporating the complex confers PRC2 partial function of the initiate silencing.^49^ Through binding to the aromatic cage of EED and remodelling the SRM domain of EZH2, JARID2 is trimethylated at lysine 116 mimics (JARID2K116me3) (Figure 3B) which has an allosteric stimulatory effect on PRC2 histone methyltransferase activity.^61^ It is noteworthy that JARID2‐K116me3 promotes the allosteric activation of PRC2 but does not participate in H3K27me3, which is invoked as one of a mechanism for PRC2 deposition to unmodified nucleosomes.^62^ When H2AK119ub1 enriches at CpG islands (CGIs), EED‐mediated JARID2 cooperation with H2AK119ub1 could contribute to the CGI preference of PRC2.^63^, ^64^ More recently, the trimethylated state of PALI1 has also been shown can allosterically activate the PRC2 complex when bound to EED through a similar mechanism to that proposed for H3K27me3 (Figure 3C,D).^65^

## 4 THE INTERACTION OF EED AND H3K27ME3

Mammalian heterochromatin contains great repressive chromatin domains, including H3K9me2/3‐modified constitutive heterochromatin and H3K27me2/3‐decorated facultative heterochromatin. The two categories of histone modifications are catalysed by Suv39h1/2 and PRC2, respectively.^66^, ^67^ PRC2 deposits the repressive histone mark H3K27me3 through an aromatic cage of EED and establishes the direct interaction with its target genes.^20^ Furthermore, PRC2 binding or catalytic activity is also affected by histone modifications in the chromatin region, one of which is H3K27me3.^68^ Aranda et al. report that EED has high‐affinity binding for histone methylations correlated with transcriptional repression.^69^ EED can bind H3K27me3 when H3K27me3 is present on two adjacent nucleosomes, and the presence of H3K27me3 stimulates PRC2 enzyme activity, thus generating a positive feedback loop. Mutations of the EED aromatic cage or EED absence will disrupt this interaction and lead to a global loss of H3K27me3.^47^, ^52^, ^70^, ^71^ Other repressive chromatin marks that can be recognized by EED, such as H3K9me3, H4K20me3 and H1K26me3, indicate that recognition of repressive histone marks via EED could serve as a mechanism of PRC2 recruitment to silenced loci.^20^, ^48^, ^72^

## 5 EED FUNCTIONS IN ONTOGENESIS

**FIGURE 4:**
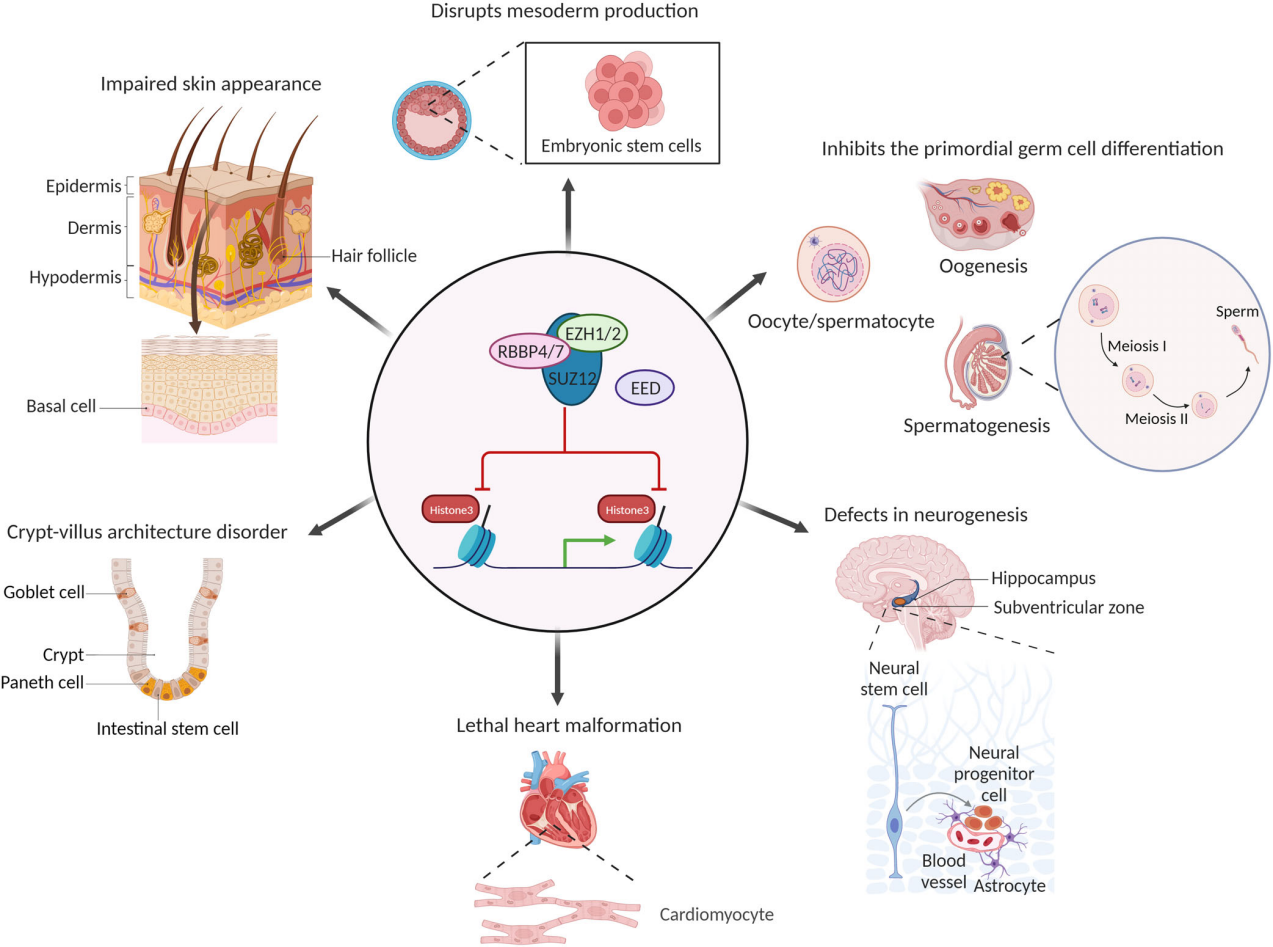
Embryonic ectoderm development (EED) protein is reside in various organ morphogenesis, including embryonic development, spermatogenesis, oogenesiscy, neurogenesis, and so on. Polycomb repressive complex 2 absent EED results in multiple developmental abnormalities.

For pluripotent stem cells to expand and differentiate into one or more specialized and committed cell types in the body, critical cell fate decisions and lineage commitment must be made during growth and development. As differentiating stem cells undergo the cascade of lineage decisions on branching points through epigenetic‐threshold modulation, specific genes must be switched on and genes associated with alternative lineages need to be repressed in a dynamic and timely manner. Next, we will dissect the dynamic roles of EED during ontogenesis to further insight into the PRC2 and its role in the specificity and diversity of lineage specification (Figure 4).

### 5.1 Embryonic development

ESCs possess the ability to differentiate into all the cell types of tissues and organs.^73^ ESCs pluripotency is governed by a gene regulatory network composed of various TFs and chromatin‐modifying enzymes.^74^ PRC2 complex is essential for early embryonic development and has been implicated in pluripotency as well as cell fate determination in ESCs. In human and murine ESCs, PRC2 localizes to the promoters of repressed genes, which encode TFs for specification later during development, and EED mutations cause their premature expression.^75^ EED is also required for regulating normal murine embryogenesis. When mice are knocked out of EED, early embryonic lethality occurs because they fail to properly gastrulate and produce embryonic mesoderm.^76^, ^77^, ^78^ Montgomery et al. demonstrate that EED^−/−^ embryos result in a genome‐wide decrease in H3K27me1, H3K27me2 and H3K27me3.^19^ ESCs absent EED results in inactivating histone methyltransferase and lack the H3K27me3 modification.^21^, ^79^ EED and H3K27 methylation are also involved in the gene regulation of undifferentiated and differentiating cells, facilitating ESCs differentiation towards a specific lineage. Through performing gene expression and chimera analyses on both low and high‐passage EED null ESCs, Chamberlain et al. identify that EED null ESCs fail to differentiate properly in vitro, but can contribute to chimeras.^80^ ESCs without EED can maintain pluripotency markers and self‐renew, but fail to execute differentiation programmes promptly.^81^ These results focus on studying undifferentiated ESCs. By contrast, Obier et al. perform global gene expression analysis in both undifferentiated ESCs and embryoid body formation of EED knocked out, and results demonstrated that EED is required to silence the pluripotency network during differentiation.^82^ Galonska et al. find both H3K27me3 and DNA methylation absence when EED‐deficient ESCs culture in the two inhibitors (2i) conditions.^83^ But the latest research shows that genome‐wide DNA methylation and H4 acetylation are increased in the EED‐deficient ESCs culture in the 2i conditions.^84^ Hence, EED is critical for ESCs as it regulates both developmental control genes and a subset of canonically imprinted genes.

### 5.2 Spermatogenesis and oogenesis

The primordial germ cells (PGCs) are precursors of the oocyte and sperm, which transmit significant epigenetic information to the offspring.^85^, ^86^, ^87^ High EED expression is concurrent with a high level of H3K27me3 enrichment in XX and XY PGCs during development.^88^, ^89^ EED is a key effector of oocyte meiosis and spermatogonia differentiation. Spermatogonia is a heterogeneous population, consisting of spermatogonia stem cells (SSCs), undifferentiated spermatogonia and differentiated spermatogonia,^90^ while EED is present in the first two of the cell population.^91^ There are also EED and H3K27me3 enriched throughout the gene body in spermatocytes, covering both introns and exons.^92^ Differential H3K27me3 enrichment at retained nucleosomes in sperm indicates that heritable epigenetic information could affect paternal offspring.^93^, ^94^ EED prevents precocious differentiation of XY and XX PGCs through responding to sex‐specific developmental signals, and EED/H3K27me3 deficient from PGCs chromatin involves itself in a synergistic pathway with H2AK119ub1 and DNA methylation to regulate the PGCs response to the niche during sex determination in the embryonic testis and ovary.^92^ By ablation of EED in the germ cells specifically, spermatocytes decrease dramatically, and eventually, no spermatids or post‐pachytene spermatocytes remain, indicating that EED is required for meiotic progression.^91^ Besides, both SSCs and undifferentiated spermatogonia markers Lin28a, Ngn3 and Nanos3 were significantly decreased in EED mutants. Loss of EED results in SSCs deficiency and abnormal meiotic chromosome dynamics, ultimately leading to male infertility. Stringer et al. highlight that sporadic sub‐fertility of lacking EED in the paternal germline produces sub‐fertile male offspring, involving de‐repression of both LINE elements and retrotransposed pseudogenes.^94^ Impressively, Oocyte‐specific deletion of EED results in H3K27me3 absent and a significant overgrowth of offspring, which involves both adiposity and bone mineral density increase.^95^ Recent research has shown that loss of EED in somatic and germ cells leads to abnormal ovaries in adult mutant females, but the mutants are fertile.^96^ Overall, epigenetic inheritance altered by EED is important to spermatogenesis and oogenesis, especially regulating repetitive sequences in the paternal germline.

### 5.3 Neurogenesis

During the development of the nervous system, the PRC2 complex plays a vital role in maintaining the self‐renewal and proliferation capacities of neural stem/progenitor cells (NSPCs).^97^ Embryonic neurogenesis is initiated in the neuroepithelial cells of the ventricular zone (VZ), and the subventricular zone (SVZ) differentiates into radial glial cells (RGCs). RGCs can either directly produce neurons or generate neuronal intermediate progenitor cells, which differentiate into neurons, astrocytes and oligodendrocytes. While in the adult brain, NSPs are in the SVZ and the subgranular zone of the hippocampal dentate gyrus (DG).^98^ EED is indispensable for spinal cord development and NSPCs proliferation in the SVZ region. Partial mutations in genes encoding the EZH2 or EED subunits lead to Weaver syndrome,^99^ characterized by variable intellectual disability and distinctive facial features. EED is highly expressed in the brain and involved in the differentiation and maturation process of the central nervous system cells. Downregulation of EED in the spinal cord and neural tube inevitably leads to spina bifida and neural tube defects.^100^ An early study has been done to characterize EED as a key regulator of neurogenesis using EED‐deficient mice.^101^ Conditional knocked out (cKO) of EED in neural progenitors of the neocortex leads to a prolonged neurogenic phase and deferring astrocytes differentiation.^97^ Sun et al. report that EED promotes TF Gata6 expression and reduces p21 protein level when EED is deleted in neural stem cells of the SVZ, indicating the importance of EED to NSPCs proliferation and neurogenesis in the SVZ.^102^ Besides, the proper formation of DG is also required for EED. In the absence of EED in the NSPCs, cyclin‐dependent kinase inhibitor 2 A (Cdkn2a) expression is increased and critical gene SRY‐box transcription factor 11 (Sox11) for neural differentiation is repressed, which ultimately leads to shorter and smaller dentate gyrus formation, indicating that EED primarily acts as an activator for maintaining proliferation and differentiation.^39^ EED is essential for oligodendrocyte (OL) remyelination, while it is dispensable for myelin maintenance. EED deletion in OL progenitors results in H3K27me3 absent and OL lineage abnormalities.^40^ Yaghmaeian and colleagues also find H3K27me3 disruption in the developing mouse hypothalamus when EED is cKO, which triggers reduced cell proliferation, ectopic expression of posteriorly expressed regulators and increased expression of cell‐cycle regulators.^103^ In addition to NSPCs and oligodendrocytes, recently, microglial EED has been demonstrated to be essential for synaptic pruning during normal postnatal brain development.^104^ Furthermore, deletion of EED in the forebrain leads to the upper‐layer neuron numbers being significantly decreased and abnormal cortical architecture. Genomic and transcriptomic network analyses indicate that abnormal acetylation of H3K27 (H3K27ac) accumulation is associated with the decrease of H3K27me3 and the recruitment of RNA‐Pol2.^41^ Consequently, EED has multiple functions in neurogenesis, and it plays dynamic roles in NSPCs maintenance and the stage of gliogenesis.

### 5.4 Cardiogenesis

The development and function of the cardiovascular system are vulnerable to epigenetic insults, one of which is epigenetic repressors PRC2.^105^, ^106^, ^107^ In the murine hearts, alterations of chromatin landscape have repeatedly been linked to both cardiomyopathy and structural heart disease in postmitotic cardiomyocytes.^37^, ^106^, ^108^, ^109^ Moreover, the inactivation of EED in murine foetal cardiomyocytes leads to cardiac fibrosis and significant systolic impairment. During early heart development, loss of H3K27me3 is complete upon cardiac‐specific inactivation of EED, resulting in lethal heart malformations.^105^ The previous study has shown that EED interacts with significant amounts of proteins in the Endothelin‐1‐induced cardiomyocyte proteome.^110^ EED also interacts with phospholipase neutral sphingomyelinase 2 (N‐SMase2) through coupling TNF‐R1 to N‐SMase2, thus mediating heart failure and atherosclerosis.^111^ Using Tie2Cre to inactivate the floxed murine EED results in absence of blood‐perfused vasculature via exhausting the haematopoietic stem cells pool.^112^ Ai et al. highlight that in EED cKO mice, significant loss of H3K27me3 can be observed, but it does not directly regulate the upregulation genes, which include upregulated skeletal muscle genes. In contrast, abnormal H3K27ac accumulation is associated with these upregulated genes, indicating that EED complexes with and stimulates HDAC deacetylase activity to silence the slow‐twitch myofiber gene programme to orchestrate heart maturation.^37^ Furthermore, with absent EED in postmitotic cardiomyocytes of adult mouse hearts, Li et al. find a long lifetime of cardiomyocytes accompanied by a short histone half‐life.^113^ Mechanistically, EED ablation is involved in histone flux and nucleosome remodelling through BRG1, which results in the inhibition of nucleosomal histones exchange and histone turnover, eventually leading to H3K27ac accumulation. Besides, EED‐knockdown in human pluripotent stem cells significantly enhanced cardiac differentiation with increasing expression of multiple cardiac genes, such as myocyte enhancer factor 2C (MEF2C), myosin heavy chain 6/7 (MYH6/7), cardiogenic TFs NKX2.5 (NKX2‐5), and so on.^114^

### 5.5 Intestinal morphogenesis

Intestinal stem cells (ISCs), located at the bottom of the crypts, are a cell population self‐renew extensively and differentiates into all cell types within crypts and villi.^34^, ^115^, ^116^, ^117^ In the developmental and adult intestine, PRC2‐mediated H3K27me3 deposition is required for transitions from ISCs to transit‐amplifying progenitors and post‐mitotic villus cells.^34^, ^116^, ^118^ It is estimated that about 2000 genes are marked by H3K27me3 in both crypt and villus cells.^119^ By cKO EED in the intestine, Chiacchiera et al. report that crypt‐villus architecture disorders attributed to EED affect cell cycle progression and differentiation of transient amplifying cells at the crypt bottom instead of ISCs maintenance.^116^ Conversely, Koppens and colleagues find EED deletion in the intestine using the same animal model results in uncommitted crypt cells in an aberrant differentiation and reduced cell proliferation.^34^ More crucially, the loss of ISCs and inhibition of Wnt signalling are also highlighted in this paper. The difference should be expected due to a compromised response to the long‐time βNF administration, which might affect the ISCs compartment. But the two studies coincide in the most striking result: Loss of EED causes inactivation of PRC2, and the cell cycle is arrested in crypts, which can be attributed to deleting and de‐repression of Cdkn2a. Surprisingly, genes essential for intestinal development are silenced by H3K27me3 in the adult intestinal epithelium but reactivated without PRC2 action, which is associated with functional interactions of H3K27me3 with H3K4me3.^120^ A novel observation reveals that the absence of EZH2 and H3K27me3 in EED‐null villi cells results in stunted and dysmorphic villi.^121^ By performing absenting EED in distinct cell compartments of the intestinal epithelium, the further investigation reveals that H3K27me3 loss occurs as a result of replicational dilution, which occurs proportional to the frequency of cell division.

### 5.6 Skin and hair follicle morphogenesis

The skin is the first barrier that protects mammals against external insults and dehydration. Epidermal lineages derive from a single layer of multipotent skin progenitors named basal cells, which attach to underlying basement membranes separating the epidermis from the dermis.^122^, ^123^ The basal cells generate the hair follicles, the sebaceous glands, the interfollicular epidermis, and the Merkel cells.^124^, ^125^ Among these, the hair follicles contain stem cells of the dermal and epidermal lineages.^126^ EED is expressed mainly in basal epidermal cells while downregulated upon differentiation during developing skin.^127^ Besides, EED and H3K27me3 are downregulated, whereas H3K27me3 demethylase UTX (lysine demethylase 6A, KDM6A) and JMJD3 (lysine demethylase 6B, KDM6B) are enriched during mice skin repair, indicating that losing polycomb‐mediated silencing may be involved in the induction of repair genes.^128^ EED‐specific deletion in the skin epithelium contributes to premature epidermal barrier development, ectopic Merkel cell formation, and postnatal hair follicle developmental hurdle.^129^ Further study indicates all hair follicle types but not just primary hair follicles, have the potential to induce Merkel cells when lacking EED in the skin epidermis.^130^ EED absence leads to the de‐repression of Sox2, Atoh1 and Isl1, the master TFs required for normal Merkel cell differentiation. Interestingly, deletion of EED decreases the proliferation of hair follicle progenitor cells rather than basal cells, indicating there may be a mechanism compensating for the loss of EED function in the epidermal progenitor cells. A recent study has provided an explanation for this phenomenon. Cohen et al. ablate both PRC1 core (Ring1a and Ring1b) and PRC2 core (EED) subunits in embryonic epidermal stem cells (EpSCs) using Krt14‐Cre mice. Neither H2AK119ub nor H3K27me3 histone can be detected in the EED; Ring1a/b 3KO epidermis.^131^ The mutant mice died shortly after birth with a visibly impaired skin appearance. Loss of both PRC1/2 complexes in EpSCs leads to the key epidermal lineage TFs such as P63 and SATB1 being significantly down‐regulated in EpSCs, indicating functional redundancy between PRC1 and PRC2 in the maintenance of epidermal stem cell identity. Moreover, EED absences in adult hair follicle stem cells (HFSCs) cannot induce HFSCs activation or fate switch.^132^ In contrast, loss of EED function in adult epidermal stem cells results in epidermal pigmentation.^133^ These studies highlight the functional complexity of EED in different cell lineages of the developing and adult epidermis.

### 5.7 Haematopoiesis

The bone marrow (BM) contains haematopoietic and mesenchymal stem cells.^134^, ^135^ Haematopoietic stem cells (HSCs) of neonatal BM self‐renew rapidly to expand the HSC pool during the growing stage.^136^, ^137^, ^138^ However, adult BM HSCs remain quiescent and demonstrate a capacity to regenerate after injury.^139^, ^140^ EED has high expression in neonatal and adult HSCs.^38^, ^138^ Haematopoiesis defects are observed in the BM and thymus of the EED mutant animals.^141^, ^142^ VavCre‐mediated EED excision appeared neonatal pale and hypocellular, leading pups dead within 5–12 days after birth.^138^ EED deletion in embryos perturbs the BM HSCs differentiation into restricted lineage progenitor cells.^112^ At the same time, loss of EED in adult BM HSCs enhances gene expression associated with proliferation and differentiation, which results in the exhaustion of HSCs.^138^ Ueda et al. generate EED mutants by knock‐in I363M to impair the EED structural integrity and find mutated‐homozygotes development arrest at E14.5.^143^ Further analysis shows that dramatically reduced H3K27me3 rather than H3K27me1/me2 or H3K4me3 occurs in EED homozygous mutants, indicating the structural integrity of EED is essential for the H3K27me3 mark. Interestingly, EED heterozygous mutants can survive the whole lifespan but undergo age‐dependent expansion and hyperproliferation of BM haematopoietic progenitors, increasing susceptibility to hematologic malignancies.^141^, ^143^, ^144^ Therefore, EED complete and partial loss‐of‐function appear to have different effects on BM haematopoiesis.

### 5.8 Osteogenesis

Bone formation involves two major distinct mechanisms: intramembranous and endochondral ossification.^145^, ^146^ Cartilage is gradually replaced by bone via endochondral ossification to form the most mammalian skeleton.^147^ Besides, mesenchymal cells that originate from the neural crest are responsible for the development of the craniofacial skeleton.^148^ De novo germline mutations in the human EED gene lead to Weaver syndrome, a disease characterized by skeletal defects, advanced bone age and overgrowth.^149^, ^150^, ^151^, ^152^ EED interacts with Bmi1 in genetic, biochemical and molecular to mainly regulate an overlapping set of Hox genes, thus avoiding developmental abnormalities of the vertebra.^153^ Absent EED in chondrocytes causes efficient elimination of H3K27me3, severely deformed thoracic spine, and shortening the long bones. In EED cKO mice, hypoxia‐inducible transcription factor 1α (Hif1a) down‐regulation induces cell death in the central area of epiphyseal growth plates. In contrast, overactivation of Wnt signalling leads to premature hypertrophic differentiation and premature growth plate closure.^35^ Moreover, H3K27me3 and H3K27me3 demethylase UTX enrichment in cartilage correlates are linked to osteoarthritis. The latest study shows that EED‐blocked expression is associated with UTX loss‐induced H3K27 hypomethylation, which showed few gonarthrotic symptoms in collagen‐induced arthritis.^154^ Collectively, EED regulates early mesenchymal lineage to differentiate towards the osteogenic lineages, thereby having a great influence on bone formation, but further investigation is need to elucidate the specific mechanism.

## 6 CONCLUSIONS

In this study, we comprehensively and systematically reviewed the research advances on EED/PRC2 function regulating ontogenesis (Table 1). PRC2 complex is the master epigenetic regulator of developmental and cell identity genes. As a core scaffold subunit of PRC2, EED plays separate and specific roles in different lineages and various time points. In this review, detailed biochemical and structural characterization of EED has expanded current views regarding how EED cooperates with other subunits forming PRC2 chromatin domains to catalyse histone modifications and engage with nucleosomes. We also have highlighted the numerous phenotypes of EED mutants during the development of different tissues and organs and summarized the general mechanisms of the role of EED in gene regulation in distinct lineage cells. Although much of our understanding of the EED has come from studies on various stem cells and specific tissue development, the mechanisms that guide EED in maintaining tissue homeostasis of adult organisms are likely to vary considerably. There is undoubtedly considerable scope for further research to better understand the function and regulation of EED in adult stem cells. Another optimization goal for future studies would be understanding the spatiotemporal‐specific changes in cellular EED levels, this will include improving our knowledge of the distinct functions of PRC2 core subunits. The variety of functions played by EED further expands the complex yet fascinating mechanisms and principles of PRC2‐mediated gene regulation during embryogenesis and organogenesis. In summary, insights provided in the present review will endow researchers concise understanding of the role of EED‐mediated epigenetic regulations in development. With the continuous improvement of molecular tools and sequencing technologies, the opportunity for future research into the dynamic role of EED, as well as its contribution to the development of organs and the progression of numerous diseases remains wide open.

**TABLE 1:** Functional regulation of embryonic ectoderm development (EED) in ontogenesis.

Ontogenesis	Regulated function	References
Embryonic development	Target genes are de‐repressed	Faust et al.^76^
	Genome‐wide decrease in H3K27me1, H3K27me2 and H3K27me3	Montgomery et al.^19^
	Decrease in Ezh2 protein levels. Disrupted axial patterning	Chamberlain et al.^80^
	Fail to properly gastrulate and to produce embryonic mesoderm	Leeb et al.^81^
	EED null ESCs fail to differentiate properly in vitro, but can contribute to chimeras	Obier et al.^82^
	EED is required to silence the pluripotency network during differentiation	van Mierlo et al.^84^
	Genome‐wide of DNA methylation and H4 acetylation are increased in the EED^−/−^ 2i ESCs	
Spermatogenesis and oogenesis	Inhibits the spermatogonia differentiation	
	Impedes meiotic progression	Mu et al.^91^
	Synergistic with H2AK119ub1 and DNA methylation	Prokopuk et al.^93^
	Male infertility	Stringer et al.^94^
	Adult mutant females are fertile	Prokopuk et al.^96^
	Prevents precocious differentiation of XY and XX PGCs	Lowe et al.^92^
Neurogenesis	A key regulator of the neurogenesis; prolonging neurogenic phase and deferring astrocytes differentiation	Schumacher et al.^101^
	Spina bifida and neural tube defects	Hirabayashi et al.^97^
	Promotes neurosphere formation	Song et al.^100^
	Regulates proliferation in the telencephalon	Sun et al.^102^
	Shorter and smaller dentate gyrus	Yaghmaeian et al.^103^
	Mediates oligodendrocyte remyelination; maintains normal synaptic and cognitive functions	Liu et al.^39^
	Controls embryonic cortical neurogenesis	Wang et al.^40^
		Wang et al.^104^
		Zhang et al.^41^
Cardiogenesis	Mediates heart failure and atherosclerosis	Philipp et al.^111^
	Lethal heart malformations	He et al.^108^
	Participates in ET‐1 induced cardiomyocyte terminal differentiation	Shin et al.^110^
	Abnormal H3K27ac accumulation	Ai et al.^37^
	Regulates lifetime of cardiomyocytes	Li et al.^113^
	Enhances cardiac differentiation	Liu et al.^114^
Intestinal morphogenesis	Crypt–villus architecture disorders	Chiacchiera et al.^116^
	Uncommitted crypt cells in an aberrant differentiation and reduced cell proliferation	Koppens et al.^34^ Jadhav et al.^120^
	Significant weight loss and severely degraded crypt	Jadhav et al.^121^
	Stunted and dysmorphic villi	
Skin and hair follicle morphogenesis	Premature epidermal barrier development, ectopic Merkel cell formation, and postnatal hair follicle developmental hurdle	Dauber et al.^129^
	Decreases the proliferation of hair follicle progenitor cells	Perdigoto et al.^130^
	Cannot induce HFSCs activation or fate switch; epidermal pigmentation	Cohen et al.^131^
		Flora et al.^132^
		Li et al.^133^
Haematopoiesis	Haematopoiesis defects	Lessard et al.^141^
	Increasing susceptibility to hematologic malignancies of EED heterozygous mutants	Majewski et al.^144^
	Neonatal pale and hypocellular	Xie et al.^138^
	Perturbs the BM HSCs differentiation into restricted lineage progenitor cells	Yu et al.^112^
		Ueda et al.^143^
Osteogenesis	Weaver syndrome	Cohen et al.^150^
	Regulates vertebra development	Cooney et al.^151^
	Causes severely deformed thoracic spine, and shortening the long bones	Kim et al.^153^ Mirzamohammadi et al.^35^
	Shows few gonarthrotic symptoms in collagen‐induced arthritis	Lian et al.^154^

## AUTHOR CONTRIBUTIONS

Liuyan Huang conceived, wrote, revised the manuscript and made the figure and table. Fanyuan Yu and Feifei Li revised the manuscript. Ling Ye, Chenglin Wang and Fanyuan Yu reviewed, revised and edited the manuscript. All authors read and approved the final manuscript.

## FUNDING INFORMATION

This work was supported by National Natural Science Foundation of China 81873708 (Chenglin Wang), 82201045 (Fanyuan Yu), Sichuan Province Science and Technology Program 2022JDRC0130 (Fanyuan Yu) and 2022ZYD0055 (Fanyuan Yu) and Young Elite Scientist Sponsorship Program by CAST 2022QNRC001 (Fanyuan Yu).

## CONFLICT OF INTEREST STATEMENT

The authors declare no potential conflicts of interest.
